# Physicochemical Characteristics, Antioxidant Properties, and Identification of Bioactive Compounds in Australian Stingless Bee Honey Using High-Performance Thin-Layer Chromatography

**DOI:** 10.3390/molecules30061223

**Published:** 2025-03-09

**Authors:** Mariana Mello dos Santos, Tomislav Sostaric, Lee Yong Lim, Cornelia Locher

**Affiliations:** 1 Centre for Optimisation of Medicines, Division of Pharmacy, School of Allied Health, The University of Western Australia, Crawley, WA 6009, Australia; mariana.mellodossantos@research.uwa.edu.au (M.M.d.S.); tom@chromatechscientific.com (T.S.); lee.lim@uwa.edu.au (L.Y.L.); 2 Institute for Paediatric Perioperative Excellence, The University of Western Australia, Crawley, WA 6009, Australia

**Keywords:** antioxidant activity, chemical profile, stingless bee honey, *Tetragonula carbonaria*, *Tetragonula hockingsi*, Australia, sugar, total phenolic

## Abstract

This study investigates the physiochemical properties, chemical composition, and antioxidant activity of Australian stingless bee honey blends from two bee species, *Tetragonula carbonaria* and *Tetragonula hockingsi*, harvested in Burpengary East, Queensland at different times of the year. The moisture content of the honey samples ranged from 26.5% to 30.0%, total soluble solids from 70.0 to 73.5° Brix, and pH from 3.57 to 4.19. The main sugars identified were trehalulose (13.9 to 30.3 g/100 g), fructose (12.9 to 32.3 g/100 g), and glucose (4.80 to 23.7 g/100 g). The total phenolic content (TPC), measured using the Folin–Ciocalteu assay, ranged from 26.1 to 58.6 mg of gallic acid equivalents/100 g. The antioxidant activity was investigated with the 2,2-diphenyl-1-picrylhydrazyl (DPPH) assay, with values ranging from 1.39 to 6.08 mmol of Trolox equivalents/kg. Antioxidant constituents were determined using a High-Performance Thin-Layer Chromatography (HPTLC)-DPPH assay. The HPTLC-DPPH analysis revealed that honey samples collected in May 2022 contained the highest number of antioxidant compounds. Some constituents were identified using an HPTLC-derived database and also quantified utilising HPTLC analysis. Lumichrome was present in all honey samples, while luteolin and kaempferide were detected only in some. Kaempferol or isorhamnetin was also found to be present, although a definitive distinction between these two chemically closely related compounds could not be made by HPTLC analysis. The results showed that honey produced by *Tetragonula hockingsi* and *Tetragonula carbonaria* shares similar properties and composition when harvested at the same time, with only minor differences in moisture, fructose, and glucose content.

## 1. Introduction

Traditional medicine has been practiced for thousands of years, with honey being one of the oldest and most valued treatments for various human diseases [1]. Honey produced by the European honeybee, *Apis mellifera,* has been extensively studied, revealing significant antibacterial and antioxidant properties. These properties are linked to anti-inflammatory, anticancer, and anti-aging effects [2].

Along with honey derived from European honeybees (*Apis mellifera),* Australia also produces stingless bee honey, known as sugarbag, which has a rich history of medicinal use among First Nations peoples. However, unlike *Apis mellifera* honey, research on the bioactivities of Australian stingless bee honey remains limited.

There are approximately 500 species of stingless bee distributed throughout the tropical and subtropical regions of the world, including Australia, Africa, Latin America, and Southeast Asia [3]. These bees play a crucial role as pollinators for native flora and also contribute significantly to the pollination of various crops [4]. Stingless bee products are considered to be superior sources of biologically active compounds, such as phenolic compounds, compared to European honeybee products. This advantage is largely attributed to the rich vegetation in the tropical and subtropical regions where stingless bees can be found [5].

Phytochemicals, particularly phenolic acids and flavonoids, may be present in only small amounts in honey, yet they significantly influence its unique flavour, appearance, and bioactivity. These compounds may exhibit a range of beneficial effects, including antioxidant, anticancer, anti-inflammatory, antibacterial, and antiviral activities [6]. Consequently, they contribute to the notable health benefits of honey. For example, research has shown a strong correlation between the phenolic contents of honey and its antioxidant capacity [7,8].

Given the sparsity of data on the benefits of Australian stingless bee honey, this study aims to determine the physicochemical properties as well as to identify and quantify constituents in stingless bee honey samples from Australia, using a High-Performance Thin-Layer Chromatography (HPTLC)-derived database of mainly phenolic compounds [9]. It also assesses the antioxidant capacity of the honeys and determines the contribution of various constituents to their overall antioxidant activity using an HPTLC-DPPH assay. In order for this study to reflect the typical characteristics of stingless bee honey from this particular region, rather than analysing individual samples, honey blends were used for the various analyses [10,11].

## 2. Results and Discussion

### 2.1. Physicochemical Parameters

The physicochemical characteristics (moisture, pH, soluble solids, and sugar profile) of the honey blends were evaluated, and the results are shown in Table 1.

The average moisture content of the honey blends was 28.0 ± 1.36%, which is higher than that typically found in *Apis mellifera* honey, where it is usually below 20% [12]. This elevated water content may be attributed to the tropical and humid environment where the stingless bees collect nectar [13]. These moisture levels are consistent with findings from other studies on stingless bee honey in various countries. For instance, Alvarez-Suarez et al. [14] reported an average moisture content of 28.62% for Cuban stingless bee honey, while Biluca et al. [13] found a moisture content of 31% for stingless bee honey from Brazil. The highest moisture content was found in the honey collected in May and produced by *Tetragonula hockingsi* (30.0 ± 0.0577%), while the lowest moisture content was observed in the honey produced by *Tetragonula carbonaria* and harvested in November (26.5 ± 0.000%). It was also noted that, when comparing *Tetragonula carbonaria* and *Tetragonula hockingsi* honey harvested in the same month, *T. hockingsi* consistently exhibited a slightly higher moisture content.

The average soluble solids content of the stingless bee honey blends analysed was found to be 72.1 ± 1.36 °Brix, with *Tetragonula hockingsi* honey collected in May (70.0 ± 0.0577 °Brix) exhibiting the lowest °Brix value, and *Tetragonula carbonaria* honey harvested in November (73.5 ± 0.000 °Brix) showing the highest. *T. carbonaria* honey consistently displayed higher °Brix values compared to *T. hockingsi* honey when harvested in the same month. °Brix values reflect the sugar content in honey and are influenced by factors such as floral sources and environmental conditions [15,16]. They are also important indicators of potential adulteration [16,17]. Due to its higher water content and lower total sugar percentage, stingless bee honey typically exhibits lower °Brix values compared to *Apis mellifera* honey, which usually has values above 75 [15,17]. The °Brix values observed in this study are consistent with previous findings for Brazilian stingless bee honey, which reported values ranging from 71.1 to 74.7 [15].

The honey blends analysed showed an acidic pH, ranging between 3.57 and 4.19. The lowest pH was observed in the sample TH-May, while the sample TH-Nov exhibited the highest value. The pH of honey can be influenced by the floral origin of the nectar collected by the bees [18]. Additionally, bees’ mandibular secretions, the concentrations of various acids, and the presence of minerals such as calcium, sodium, and potassium can also affect pH [18,19]. The average pH of all blends analysed was 3.87 ± 0.244, which is consistent with pH values reported for stingless bee honey from Brazil (3.80) [13], Malaysia (3.29) [20], and Thailand (3.60) [3].

Carbohydrates, in the form of simple sugars, are the dominant components of honey [21]. Traditionally, fructose and glucose have been recognised as the main sugars in honey [22]. However, recent studies have identified trehalulose as a significant sugar present in stingless bee honey [23]. As shown in Table 1, there were notable statistical differences in carbohydrate levels among the stingless bee honey blends analysed. The fructose contents ranged from 12.9 g/100 g to 32.3 g/100 g, with an average of 24.9 ± 7.27 g/100 g. The glucose levels varied between 4.80 g/100 g and 23.7 g/100 g, with a mean value of 15.4 ± 7.74 g/100 g. Trehalulose was detected in all samples, ranging from 13.9 g/100 g to 30.3 g/100 g, with an average of 19.6 ± 6.52 g/100 g. Interestingly, both honey samples harvested in May exhibited the highest trehalulose levels, while also having the lowest concentrations of fructose and glucose. This suggests a potential seasonal variation in the sugar composition of stingless bee honey. *Tetragonula hockingsi* also exhibited higher fructose and glucose levels than *Tetragonula carbonaria* when harvested in the same month.

### 2.2. Compound Identification

Compounds were identified using blended honey samples rather than individual ones, as this provided a more accurate representation of the honey’s typical chemical composition. Multivariate data analysis was employed to examine the obtained HPTLC-derived fingerprints of individual honey samples and to detect patterns, helping to determine the best blending strategy [10]. The results showed a strong correlation between the fingerprints and the respective harvest months of the samples.

**Figure 1:**
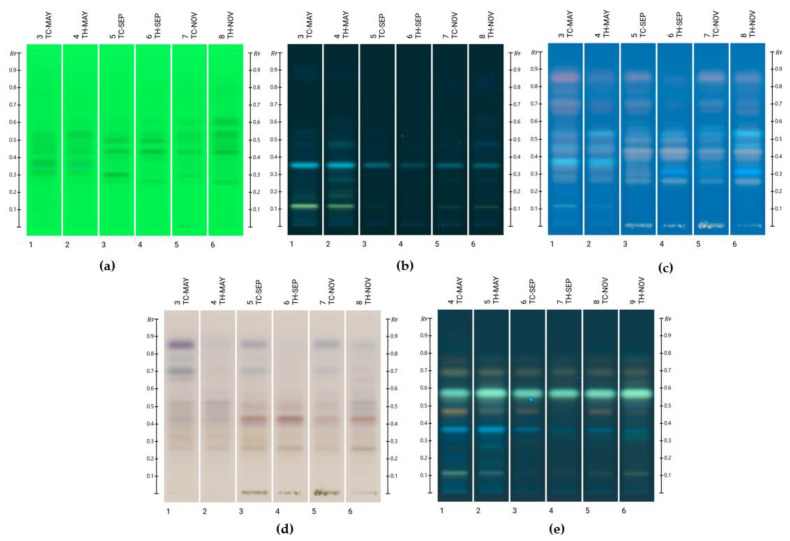
HPTLC fingerprints of honey blends using the mobile phase toluene/ethyl acetate/formic acid (6:5:1) at (**a**) 254 nm prior to derivatisation, (**b**) 366 nm prior to derivatisation, (**c**) 366 nm after derivatisation with VSA, (**d**) white light after derivatisation with VSA, and (**e**) 366 nm after derivatisation with NP-PEG.

**Figure 2:**
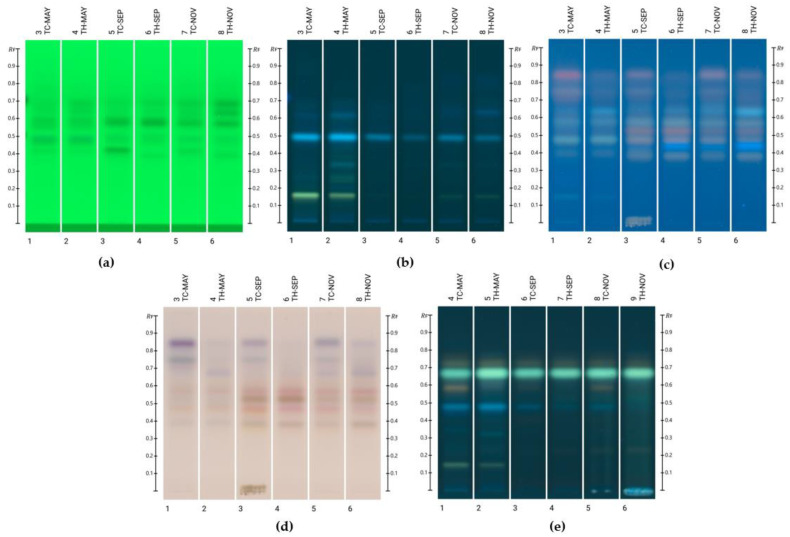
HPTLC fingerprints of honey blends using the mobile phase toluene/ethyl acetate/formic acid (2:8:1) at (**a**) 254 nm prior to derivatisation, (**b**) 366 nm prior to derivatisation, (**c**) 366 nm after derivatisation with VSA, (**d**) white light after derivatisation with VSA, and (**e**) 366 nm after derivatisation with NP-PEG.

Unknown bands in the honey blends were identified using an HPTLC database developed by Lawag et al. [9]. Rf and RGB values, along with fluorescence and UV-Vis spectra of bands of interest (absorbance (AU) > 0.05), were recorded and compared to the database’s standards. The HPTLC fingerprints generated using the two mobile phases, toluene/ethyl acetate/formic acid (6:5:1 and 2:8:1), are presented in Figure 1 and Figure 2, respectively.

Initially, the mobile phase toluene/ethyl acetate/formic acid (6:5:1) was employed to screen for potential hits. Once these were identified, the second mobile phase—toluene/ethyl acetate/formic acid (2:8:1)—was used to confirm the identification of the compounds. 

**Figure 3:**
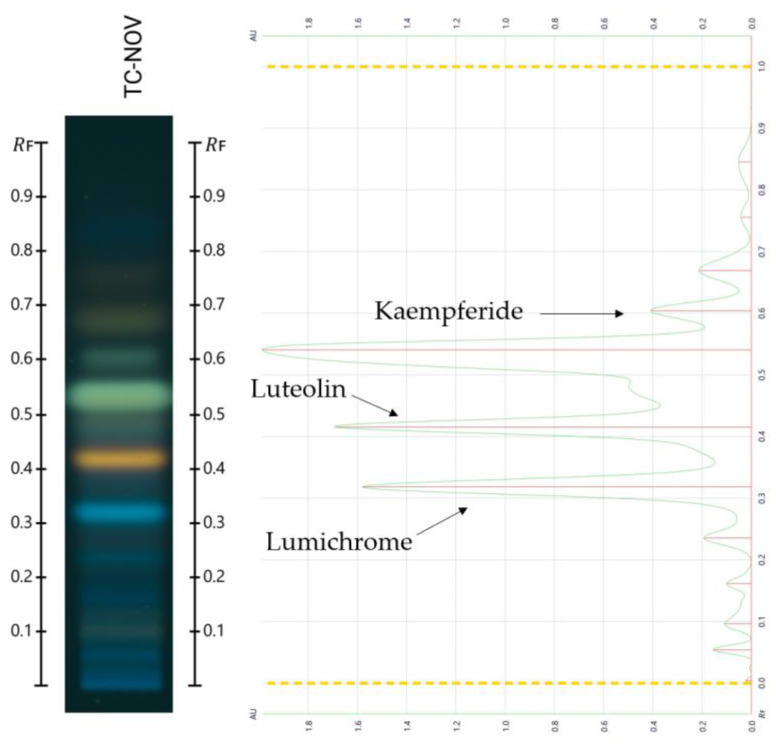
HPTLC chromatogram of the sample TC-Nov obtained with the mobile phase toluene/ethyl acetate/formic acid (6:5:1) after derivatisation with NP-PEG at 366 nm.

**Figure 4:**
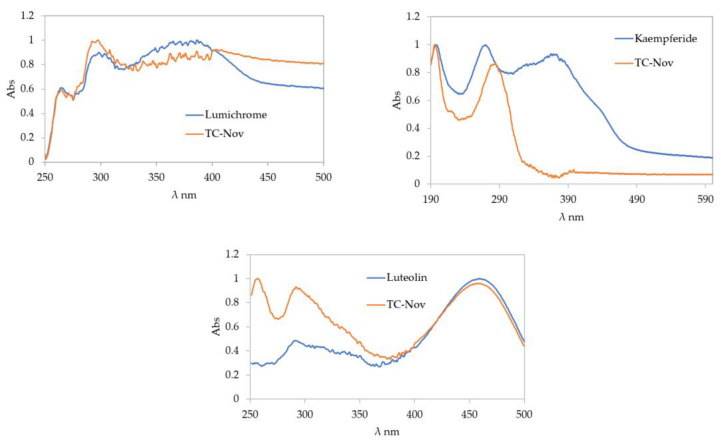
Spectral overlay of unknown bands in the sample TC-Nov using the mobile phase toluene/ethyl acetate/formic acid (6:5:1).

**Figure 5:**
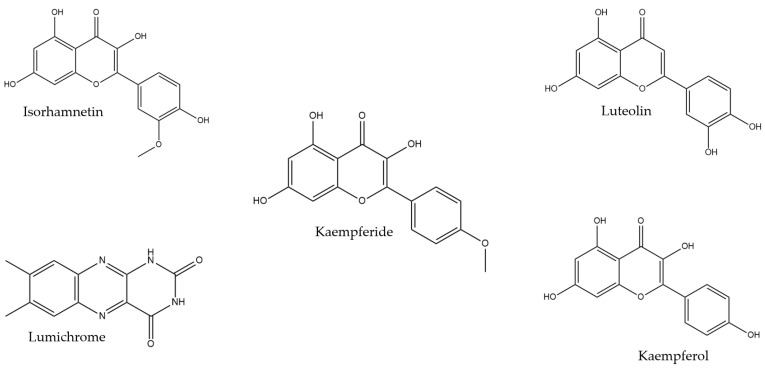
Chemical structure of the compounds identified in Australian stingless bee honey (generated using ChemDraw version 23.1.2, PerkinElmer Informatics, Inc., Waltham, MA, USA).

Following the validated screening approach developed for the HPTLC database, the following compounds were identified with the mobile phase toluene/ethyl acetate/formic acid (6:5:1): lumichrome (Rf 0.32), luteolin (Rf 0.42), and kaempferide (Rf 0.61). For another band at Rf 0.53, two possible matches—kaempferol and isorhamnetin—were found. However, due to their very similar Rf values, RGB values, and λ_max_, distinguishing between them using this HPTLC-based methodology was not possible. Further analyses using different mobile phases or alternative analytical techniques, such as liquid chromatography–mass spectrometry, would be required. Figure 3 and Figure 4 present the chromatograms and spectral overlays, respectively, for the sample TC-Nov. Chromatograms for the other investigated honey blends are provided in the Supplementary Materials (Figure S1). Figure 5 shows the chemical structures of all matched compounds.

Bioactive compounds in honey play a significant role in its health benefits. For example, lumichrome, which has also been found in Australian stingless bee honey from New South Wales [24], has demonstrated anticancer properties [25]. Luteolin has also been identified in stingless bee honey from Brazil [26,27], Cuba [14], and Ecuador [17]. This flavonoid is recognised for its anticancer effects, promoting apoptosis, inducing cell-cycle arrest, and inhibiting metastasis and angiogenesis in various cancer cell lines, including breast, colon, pancreatic, and lung cancers, among others [28,29]. Additionally, luteolin has been shown to possess anti-photoaging and anti-inflammatory properties, aiding in the reduction of psoriasis [30]. Kaempferide exhibits a range of pharmacological effects, including antitumour and anti-inflammatory activity, oxidative stress relief, and anti-hypertension properties [31,32,33].

Although the fingerprints of the honey blends revealed several bands, only a few could be confirmed using the database. This may be because the database was established based mainly on phenolic compounds found in European bee honey, which might not necessarily be present to the same extent in stingless bee honey. In fact, the limited number of matches against the database supports the theory that stingless bees access different flora, resulting in a unique constituent profile distinctly different to that of European bee honey [5,34].

### 2.3. Compound Quantification

Table 2 summarises the quantification of the identified compounds in the respective stingless bee honey blends. Lumichrome was detected in all honey blends, with its concentration ranging from 0.786 µg/g in TC-Nov to 3.99 µg/g in TH-May. Luteolin was also present in all samples except for TH-Sep, with the highest concentration observed in TC-May (1.01 µg/g) and the lowest in TH-Nov (0.338 µg/g). Kaempferide was found only in TH-May, TH-Sep, TC-Nov, and TH-Nov, with levels ranging from 0.387 µg/g in TH-Sep to 1.97 µg/g in TH-May.

This variation in the composition of the stingless bee honey samples can be attributed to several factors. Plants produce nectar that is used for honey production, and it is likely that bioactive nectar compounds that are synthesised are transferred into the honey. Therefore, the source of the nectar can influence the honey’s chemical composition. These compounds act as protective agents for plants against environmental stressors such as temperature fluctuations, light exposure, water content, UV radiation, and mineral nutrient deficiencies. As a result, variations in these compounds can occur due to the plant’s exposure to different environmental conditions, resulting in changes in its nectar composition over time and, with them, changes in the chemical profile of the honey [35,36].

Moreover, different bee species might have their own floral preferences, which will also cause variations in the chemical profile of the honey [36].

### 2.4. Total Phenolic Content and Antioxidant Activity

Stingless bee honey is recognised as a rich source of bioactive compounds, particularly phenolics, which play a key role in its antioxidant properties [37]. Table 3 shows the total phenolic content (TPC) and DPPH radical scavenging activity of the honey blends analysed.

The TPC in the six honey blends ranged from 26.1 to 58.6 mg of gallic acid equivalents (GAE)/100 g, with an average of 37.7 ± 12.0 mg of GAE/100 g. These findings confirm that stingless bee honey contains significant levels of phenolics, although these levels may vary due to factors such as the stingless bee species involved in its production, environmental conditions, flower availability, and climate [37].

The DPPH radical scavenging activity ranged from 1.39 to 6.08 mmol TE/kg, with an average of 3.48 ± 1.67 mmol TE/kg. A strong correlation (0.971) was observed between DPPH antioxidant activity and TPC, confirming the important role of phenolic compounds in the antioxidant capacity of stingless bee honey.

### 2.5. HPTLC-DPPH Antioxidant Activity

While the total DPPH assay is widely used to assess the overall antioxidant activity of natural products, its primary advantage when used in combination with HPTLC analysis is the ability to identify which specific constituents contribute to the honey’s total antioxidant activity. In brief, in this method, the RGB value of each band, generated automatically by the HPTLC software (visionCats v3.1, CAMAG, Muttenz, Switzerland) after the application of the DPPH reagent, is converted into an individual hue value. The thus-obtained hue values are used to semi-quantitatively express the radical scavenging activity of individual bands with reference to the respective activity of a gallic acid standard solution [2,38] (Table 4).

**Figure 6:**
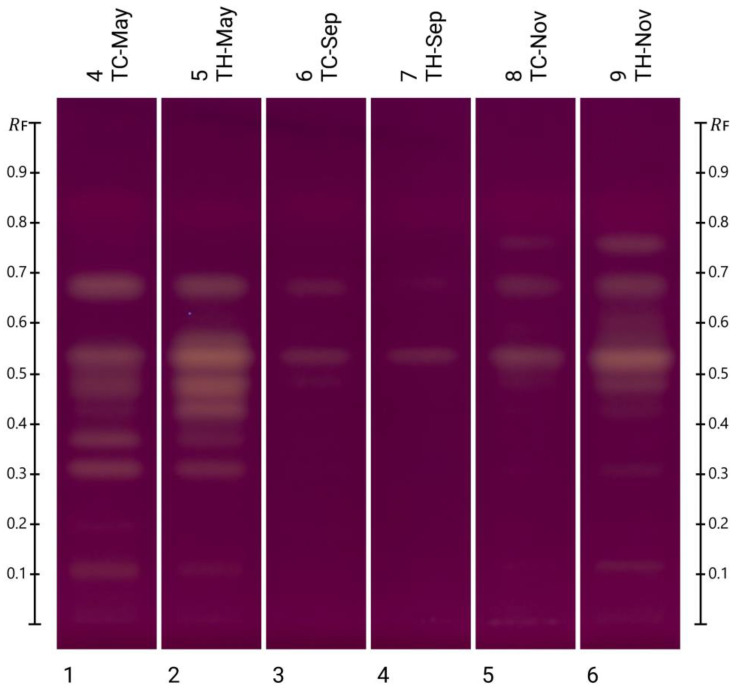
HPTLC-DPPH plate image of all honey blends.

Figure 6 shows the fingerprints obtained after HPTLC-DPPH analysis of the various honey blends. As previously mentioned, DPPH has a dark purple colour that changes to yellow upon exposure to antioxidant constituents. The intensity of the resulting yellow band is thus a reflection of the antioxidant strength of the respective honey extract constituent.

None of the samples showed bands with ‘high’ activity, defined as 66.7–100.0% RSA, but some bands with radical scavenging activity between 33.4 and 66.6% exerted ‘medium’ antioxidant activity. Interestingly, it was found that samples from both bee species collected in May exhibited the highest number of bands with antioxidant activity, which is consistent with the findings of this study’s TPC determination and the DPPH assay to capture total antioxidant activity. The May samples had the highest total phenolic contents and showed the strongest antioxidant activity in the DPPH assay, reinforcing the link between phenolic compounds and antioxidant properties. It could also be observed that all samples seemed to share two common antioxidant bands, with Rf values around 0.54 and 0.68. While two of the identified constituents, kaempferide (Rf 0.61) and luteolin (Rf 0.42), did not seem to contribute any notable antioxidant activity to the investigated honey blends, the contribution of lumichrome (Rf 0.31) could be semi-quantitatively assessed as ‘low’ (0–33% RSA) using HPTLC-DPPH analysis in the honey blends harvested in May. The two constituents contributing the highest antioxidant activity (Rf 0.48 and Rf 0.54) remain chemically unidentified, although, as mentioned earlier in Section 2.2 of this study, the compound at Rf 0.54 is likely to be either kaempferol or isorhamnetin, but further analysis is required to confirm the exact identity of this highly antioxidant constituent.

## 3. Materials and Methods

### 3.1. Chemicals and Reagents

The chemicals and reagents were obtained from the following suppliers: trehalulose (Biosynth Carbosynth, Staad, Switzerland); 2-aminoethyl diphenylborinate, fructose, sucrose, ethanol, and sodium carbonate anhydrous (Chem-Supply Pty Ltd., Port Adelaide, SA, Australia); anhydrous sodium acetate, glucose, gallic acid, and phosphoric acid (Ajax Finechem Pvt Ltd.s., Cheltenham, VIC, Australia); boric acid (Pharma Scope, Welshpool, WA, Australia); maltose, Trolox, Folin–Ciocalteu Phenol reagent 2N, and vanillin (Sigma-Aldrich, St. Louis, MO, USA); diphenylamine (the British Drug Houses Ltd., London, UK); aniline (Fluka AG, Buchs, Switzerland); 2,2-diphenyl-1-picrylhydrazyl (DPPH) (Thermo Fisher Scientific, Ward Hill, MA, USA); toluene (APS Chemicals, Sydney, NSW, Australia); 4,5,7-trihydroxyflavanone (Alfa Aesar, Lancashire, UK); ethyl acetate, formic acid and sulfuric acid 98 % (Ajax Finechem, Wollongong, NSW, Australia); polyethylene glycol 400 (PharmAust, Welshpool, WA, Australia); kaempferide (Angene International Ltd., Nanjing, China); kaempferol and luteolin (Combi-Blocks Inc., San Diego, CA, USA); lumichrome (Sigma Aldrich, Castle Hill, NSW, Australia); isorhamnetin (Wuhan ChemFaces Biochemical, Wuhan, Hubei, China); and methanol and dichloromethane (Merck KGaA, Darmstadt, Germany).

Silica gel 60 F254 HPTLC glass plates (20 cm × 10 cm) were sourced from Merck KGaA (Darmstadt, Germany).

### 3.2. Honey Samples

Thirty-two stingless bee honey samples were directly sourced from a local beekeeper in Burpengary East, Queensland, Australia (Table 5). The samples, collected from two bee species—*Tetragonula carbonaria* (*n* = 26) and *Tetragonula hockingsi* (*n* = 6)—were harvested in May (*n* = 12), September (*n* = 10), and November (*n* = 10) of 2022 and stored at 4 °C until use.

### 3.3. Sample Preparation

To better represent typical constituent patterns and characteristics, honey blends were prepared. For this, the HPTLC fingerprints of individual honey samples were analysed and, based on the results of multivariate data analysis of these fingerprints [10], pooled samples were prepared by mixing equal amounts of individual samples obtained from the same bee species and at the same harvest period, resulting in a final weight of 10 g of the respective blend, as shown in Table 6. All subsequent analyses were conducted using the honey blends rather than the individual samples.

### 3.4. Physicochemical Analysis

#### 3.4.1. Soluble Solids (°Brix) and Moisture Content

To measure the total soluble solids in the honey blends, a small amount of each sample, at room temperature, was placed on the prism of a digital refractometer (Hanna Instruments, HI96801, Woonsocket, RI, USA). The results, expressed in °Brix [39], were used to calculate the honey moisture content as (100%—°Brix).

#### 3.4.2. pH

The pH of the honey solution was measured at room temperature using a pH meter (Oakton, pH 700, Singapore). The testing solution was prepared by dissolving 1 g of honey in 7.5 mL of deionised water [39].

#### 3.4.3. Sugar Profile

For the determination and quantitative analysis of the main sugars present in the honey samples, High-Performance Thin-Layer Chromatography (HPTLC) was used. To quantify the main sugars, standard solutions of trehalulose (200 µg/mL), glucose (250 µg/mL), and fructose (250 µg/mL) were prepared in 50% aqueous methanol, and calibration curves were generated for trehalulose (100–800 ng/band), fructose (250–1250 ng/band), and glucose (250–1250 ng/band).

Honey solutions were prepared at 1 mg/mL concentrations in 50% aqueous methanol and separated with a mobile phase consisting of 1-butanol, 2-propanol, and aqueous boric acid (5 mg/mL) at a 30:50:10 volume ratio [40,41].

Both standards and honey solutions (1 to 5 µL) were applied to the HPTLC plates using a semi-automated applicator (Linomat 5; CAMAG, Muttenz, Switzerland) at 40 nL/s, with bands placed 8.0 mm from the bottom and 20.2 mm from the side edges of the plate. The bands measured 8.0 mm, with 11.4 mm gaps between them.

The plates were developed over a distance of 85 mm using 10 mL of the mobile phase in a saturated and activated automated development chamber (ADC2, CAMAG, Muttenz, Switzerland), at 33% relative humidity and room temperature. The chamber was saturated for 60 min, followed by 5 min pre-conditioning of the plates with the mobile phase.

After development with the mobile phase, the plates were dried for 5 min. To enhance band visualisation, 2 mL of derivatisation reagent was applied. The reagent was prepared by dissolving 2 g of diphenylamine and 2 mL of aniline in 80 mL of methanol, followed by the addition of 10 mL of 85% phosphoric acid, and then diluted to a final volume of 100 mL with methanol. The reagent was sprayed using a TLC derivatiser (CAMAG Derivatiser, Muttenz, Switzerland) (yellow nozzle—level 5).

After spraying, the plates were heated at 115 °C for 10 min using a TLC Plate Heater III (CAMAG, Muttenz, Switzerland) and allowed to cool to room temperature. Once cooled, images of the plates were taken under transmission white (T-white) light [40,41] with the HPTLC imaging device (TLC Visualiser 2, CAMAG, Muttenz, Switzerland) and analysed using HPTLC software (visionCATS v3.1, CAMAG, Muttenz, Switzerland).

The analysis was carried out in triplicate, and the results were expressed as the mean sugar content (in grams) per 100 g of honey ± standard deviation.

### 3.5. Identification of Compounds in Honey

For the identification of compounds in the stingless bee honey blends, a validated screening approach against an HPTLC-derived database was adopted for all bands of interest [2,9]. For this, organic extracts needed to be prepared by dissolving approximately 1 g of each honey blend in 2 mL of deionised water, followed by three extractions using 5 mL of dichloromethane. After extraction, the combined organic layers were evaporated at 35 °C and kept at 4 °C until analysis. Before HPTLC analysis, the organic extracts were re-dissolved in 100 µL of dichloromethane. A standard reference solution of 4,5,7-trihydroxyflavanone (0.5 mg/mL in methanol) was used for comparison during the analysis.

The chromatographic separation was conducted using two different mobile phase compositions: mixtures of toluene, ethyl acetate, and formic acid at 6:5:1 (*v/v/v*) and at 2:8:1 (*v/v/v*) [39]. The HPTLC plate was then prepared by applying 5 µL of the organic honey extracts and 4 µL of the reference standard in 8 mm bands, positioned 8 mm from the edge of the plate. These applications were carried out using a semi-automated HPTLC system (Linomat 5, CAMAG, Muttenz, Switzerland) at a rate of 150 nL/s. The plates were subsequently developed in a saturated and activated (33% relative humidity) automated chamber (ADC2, CAMAG, Muttenz, Switzerland) at room temperature, with the separation progressing up to a distance of 70 mm. Once developed, the plates were visualised using a TLC Visualiser 2 (CAMAG, Muttenz, Switzerland) under three different conditions: 254 nm, 366 nm, and white light.

Derivatisation reagents were applied to enhance the bands’ visibility, with vanillin–sulfuric acid (VSA) and natural product–PEG (NP-PEG) employed for this purpose. For NP-PEG derivatisation, the plates were first sprayed with 3 mL of 1% NP reagent using a green nozzle (level 3) and dried at 40 °C for 5 min on a TLC Plate Heater III (CAMAG, Muttenz, Switzerland). They were then sprayed with 5% PEG reagent using a blue nozzle (level 2), dried again at 40 °C for 5 min, and subsequently the image was taken at 366 nm. Preparation of the NP reagent involved dissolving 1 g of 2-aminoethyl diphenylborinate in 100 mL of methanol, while the PEG reagent was made by dissolving 5 g of PEG 400 in 100 mL of ethanol (96%). For VSA derivatisation, 3 mL of 1% vanillin–sulfuric acid was applied using a yellow nozzle (level 3), with the plates heated at 115 °C (TLC Plate Heater III, CAMAG, Muttenz, Switzerland) for 3 min and allowed to cool for 2 min before visualisation at 366 nm and under white light. The VSA reagent was prepared by dissolving 1 g of vanillin in 100 mL of ethanol (96%), followed by the slow addition of 2 mL of sulfuric acid (98%).

Images of the chromatographic plates were taken using the TLC Visualiser 2 (CAMAG, Muttenz, Switzerland) and analysed using HPTLC software (visionCATS v3.1, CAMAG, Muttenz, Switzerland). Additional scans were performed on the TLC Scanner 4 (CAMAG, Muttenz, Switzerland) in UV-Vis mode (190–900 nm) and fluorescence mode (190–380 nm), both after development and following derivatisation with each reagent. The data generated, such as Rf values, colour hues, and λ_max_ in both UV-Vis and fluorescence modes, were matched against 107 standards in the HPTLC-derived database, following a validated screening protocol. Spectral overlay analysis was conducted to confirm potential matches [9].

### 3.6. Quantification of Identified Compounds in Honey

The quantification of compounds in the honey blends employed the same chromatographic methods and sample preparation as described in Section 3.5 [2]. Calibration curves were established for each standard by preparing methanolic solutions at a concentration of 100 µg/mL. The volume of honey sample applied, ranging from 4 to 27 µL, was adjusted to ensure that the compound concentrations were within the calibration curve range.

Compounds were quantified at their specific λ_max_ using the evaluation feature of the VisionCATS software (v3.1, CAMAG, Muttenz, Switzerland). The analysis was carried out in triplicate, and the results were reported as the average amount of compound (in µg) per 1 g of honey ± standard deviation.

### 3.7. Antioxidant Activity

#### 3.7.1. Total Phenolic Content (TPC)

The total phenolic contents in the honey blends were assessed using the colorimetric Folin–Ciocalteu method, based on a published methodology [42], with slight modifications. To prepare an artificial honey solution, 21.63 g of fructose, 18.13 g of glucose, 1.00 g of maltose, and 0.75 g of sucrose were mixed with 8.50 g of water [43]. A 40% (*w/v*) aqueous solution of the artificial honey was then prepared. A standard curve was created by mixing 100 µL of gallic acid solutions, with concentrations ranging from 0.06 mg/mL to 0.18 mg/mL, with an equal volume of the 40% (*w/v*) artificial honey solution.

For the assay, aliquots of 200 µL of an aqueous honey solution (20% *w/v*) were mixed with 1 mL of diluted Folin–Ciocalteu reagent (prepared by diluting 1 mL of the reagent with 30 mL of deionised water) and 800 µL of a 0.75% Na_2_CO_3_ solution. The mixture was left to incubate in the dark for 2 h. Absorbance measurements were taken at 760 nm using a spectrophotometer (Agilent Technologies, Cary 60 UV-Vis Spectrophotometer, Santa Clara, CA, USA). Meanwhile, 100 µL of deionised water spiked with 100 µL of artificial honey (40% *w/v*) was used as a blank.

The total phenolic content was calculated using the formula provided in Equation (1), and all analyses were performed in triplicate. The results were expressed as the mean gallic acid equivalents (GAE) per 100 g of honey, alongside the corresponding standard deviation.

(1) TPC(mgGAE)=(ΔAbs−intercept)/slope

#### 3.7.2. Total DPPH (2,2-Diphenyl-1-Picrylhydrazyl) Radical Scavenging Assay

The antioxidant capacity of the honey samples was assessed using the DPPH radical scavenging assay, following the protocol described in [44], including some modifications (as described in [2]).

Honey samples were dissolved in water at a concentration of 20% (*w/v*), and 10 µL of each solution was mixed with 290 µL of a DPPH reagent mixture in a 96-well microplate (Greiner Bio-One 96-well Microplate Flat Bottom). The DPPH reagent was prepared by mixing a 0.130 mM methanolic solution of DPPH with an aqueous acetate buffer (100 mM, pH 5.5) at a 19:10 (*v/v*) ratio. Trolox standard solutions, ranging from 100 to 600 µM, were prepared after adjusting their pH to 7.0 and used to construct the assay’s calibration curve.

The reaction mixture was left in the dark for 2 h, after which absorbance was recorded at 520 nm using a POLARstar Optima Microplate Reader (BMG Labtech, Allmendgrün, Ortenberg, Germany). The DPPH antioxidant activity was calculated using Equation (2) as follows:

(2) DPPH(µM Trolox)=(ΔAbs−intercept)/slope

Each sample was analysed in triplicate, with the average DPPH activity reported as mmol of Trolox equivalents (TE) per kg of honey ± standard deviation.

#### 3.7.3. HPTLC-DPPH Analysis

For the HPTLC-DPPH analysis, the same chromatographic conditions and honey sample preparation as outlined in Section 3.5 were employed. A volume of 7 µL of each honey blend extract, along with 2 µL of a methanolic gallic acid solution (0.5 mg/mL), was applied to the HPTLC plates.

The chromatographic separation was performed using a mobile phase of toluene, ethyl acetate, and formic acid (6:5:1, *v/v/v*). To enhance the bands’ visualisation, the plates were sprayed with 3 mL of a 0.4% DPPH solution in an ethanol/methanol mixture (1:1 *v/v*), using a green nozzle set to level 3 [2,45,46]. The plates were then left in the dark for 2 h. Images of the plates were recorded under white light, and the bands were scanned at 517 nm.

The colour of each band was recorded as an RGB value and subsequently converted into hue values to semi-quantitatively assess the antioxidant activity of each band [9].

Gallic acid was used as a positive control, resulting in a maximum hue value of 40° (yellow colour). The obtained hue values were used to calculate the DPPH radical scavenging activity (% DPPH RSA) of bands of interest using a previously reported equation (Equation (3) [9,47]:

(3) $$\%DPPHRSA=\left(\frac{H{}^{\circ}S+∆H{}^{\circ}P}{∆H{}^{\circ}P+H{}^{\circ}G}\right)\times 100$$

where H°G is the hue value of gallic acid, ΔH°P is the hue value of unreacted DPPH on the plate (*n* = 10), and H°S is the hue value of the band of interest. The respective band’s % DPPH RSA was then categorised as indicated in Table 7.

### 3.8. Statistical Analysis

One-way analysis of variance (ANOVA) followed by Tukey’s post hoc test was used to compare the means, when applicable. Differences between means at the 95% (*p* < 0.05) confidence level were considered statistically significant. The IBM SPSS Statistical Package (v29.0.0.0) was used to conduct the statistical analyses.

## 4. Conclusions

This study investigated the physicochemical properties, chemical composition, and antioxidant activity of Australian stingless bee honey from two species, *T. carbonaria* and *T. hockingsi*, across different times of the year. The results indicated an acidic pH for all samples, with higher moisture content and lower °Brix values compared to *Apis mellifera* honey. The sugar profile revealed trehalulose, fructose, and glucose as the main sugars, with the samples collected in May showing the highest trehalulose levels and the lowest fructose and glucose levels. The findings of this study also demonstrate that the *Tetragonula hockingsi* honeys had slightly higher moisture contents, as well as fructose and glucose levels, compared to *Tetragonula carbonaria* honeys when harvested in the same month. However, overall, both types of honey were found to have very similar physicochemical properties. Using an HPTLC-based database of 107 mainly phenolic compounds, lumichrome was identified in all honey samples, while luteolin and kaempferide were detected with varying prevalence. Either kaempferol or isorhamnetin could also be detected, although a final differentiation between the two was not possible with the chosen analytical approach. The limited number of identified compounds in this study was likely due to the database’s focus on constituents identified in European bee-derived honey, which highlights the need for a more targeted approach to stingless bee honey. Additionally, HPTLC-DPPH analysis revealed that honeys produced in May had higher numbers of compounds contributing to antioxidant activity. These samples also had the highest total phenolic contents and exhibited the strongest antioxidant activity in the DPPH total antioxidant activity assay, reinforcing the correlation between phenolic compounds and antioxidant properties, and highlighting the influence of harvest time on bioactivity. This study offers valuable insight into the unique composition of Australian stingless bee honey, and it is the first to use HPTLC analysis for identifying and quantifying compounds in these honey samples. However, further research is necessary to chemically identify more of the still-unknown constituents in these honeys, particularly those that contribute significantly to its antioxidant activity, and to expand the understanding of their bioactive potential.

## Figures and Tables

**Table 1:** Physicochemical parameters of the honey blends.

Honey Blend	pH	Moisture (%w/w)	Soluble Solids (°Brix)	Trehalulose (g/100 g)	Fructose (g/100 g)	Glucose (g/100 g)
TC-May	3.73 ± 0.0265 ^a^	27.0 ± 0.153 ^a^	73.0 ± 0.153 ^a^	30.3 ± 1.41 ^a^	12.9 ± 0.666 ^a^	4.80 ± 0.380 ^a^
TH-May	3.57 ± 0.0451 ^b^	30.0 ± 0.0577 ^b^	70.0 ± 0.0577 ^b^	24.3 ± 0.611 ^b^	19.9 ± 0.265 ^b^	7.29 ± 0.216 ^b^
TC-Sep	3.77 ± 0.00577 ^a^	28.0 ± 0.0577 ^c^	72.0 ± 0.0577 ^c^	18.8 ± 0.888^c^	25.8 ± 0.961 ^c^	15.8 ± 0.231 ^c^
TH-Sep	4.14 ± 0.0100 ^c^	29.1 ± 0.0577 ^d^	70.9 ± 0.0577 ^d^	14.4 ± 0.395 ^d^	30.1 ± 0.650 ^d^	20.5 ± 0.420 ^d^
TC-Nov	3.93 ± 0.0208 ^d^	26.5 ± 0.000 ^e^	73.5 ± 0.000 ^e^	13.9 ± 0.784 ^d^	28.5 ± 0.309 ^d^	20.5 ± 0.307 ^d^
TH-Nov	4.19 ± 0.0265 ^c^	27.1 ± 0.0577 ^a^	72.9 ± 0.0577 ^a^	15.8 ± 1.21 ^d^	32.3 ± 0.530 ^e^	23.7 ± 1.18 ^e^

Values are expressed as the mean ± standard deviation. Different superscript letters in the same column denote significant differences (ANOVA, *p* < 0.05).

**Table 2:** Quantification of identified compounds in the honey blends (µg/g, *n* = 3).

Honey Sample	Lumichrome	Luteolin	Kaempferide
TC-May	3.74 ± 0.479 ^a^	1.01 ± 0.159 ^a^	ND
TH-May	3.99 ± 0.659 ^a^	0.631 ± 0.0425 ^b,c^	1.97 ± 0.052 ^a^
TC-Sep	0.830 ± 0.0258 ^b^	0.576 ± 0.0635 ^b^	ND
TH-Sep	1.20 ± 0.166 ^b^	ND	0.387 ± 0.0211 ^b^
TC-Nov	0.786 ± 0.172 ^b^	0.804 ± 0.0827 ^c^	0.522 ± 0.0652 ^c^
TH-Nov	1.37 ± 0.170 ^b^	0.338 ± 0.0462 ^d^	0.875 ± 0.0795 ^d^

Values are expressed as the mean ± standard deviation. Different superscript letters in the same column denote significant differences (ANOVA, *p* < 0.05). ND—not detected.

**Table 3:** Total phenolic content (TPC) and DPPH radical scavenging assay of the Australian stingless bee honey blends.

Honey Blend	TPC (mg GAE/100 g)	DPPH (mmol TE/Kg)
TC-May	44.7 ± 0.861 ^a^	4.57 ± 0.282 ^a^
TH-May	58.6 ± 3.47 ^b^	6.08 ± 0.105 ^b^
TC-Sep	29.5 ± 0.429 ^c,d^	2.49 ± 0.131 ^c^
TH-Sep	26.1 ± 0.662 ^c^	1.39 ± 0.282 ^d^
TC-Nov	33.8 ± 1.60 ^d^	3.59 ± 0.0849 ^e^
TH-Nov	33.2 ± 1.70 ^d^	2.73 ± 0.0366 ^c^

Values are expressed as the mean ± standard deviation. Different superscript letters in the same column denote significant differences (ANOVA, *p* < 0.05).

**Table 4:** Percentage DPPH RSA antioxidant activity of individual bands.

Sample	Rf	Compound Match	Hue Value [°]	%RSA	Category
TC-May	0.113	-	329.6	14.9	+
	0.315	Lumichrome	335.1	21.5	+
	0.375	-	330.9	16.5	+
	0.477	-	333.4	19.5	+
	0.543	-	336.2	22.9	+
	0.683	-	338.6	25.8	+
TH-May	0.115	-	323.4	7.41	+
	0.311	Lumichrome	331.8	17.6	+
	0.374	-	325.3	9.70	+
	0.448	-	342.5	30.5	+
	0.480	-	352.4	42.5	++
	0.539	-	359.0	50.4	++
	0.580	-	332.5	18.4	+
	0.684	-	334.7	21.1	+
TC-Sep	0.541	-	328.1	13.1	+
	0.684	-	321.7	5.35	+
TH-Sep	0.545	-	324.1	8.25	+
TC-Nov	0.546	-	330.5	16.0	+
	0.681	-	326.3	10.9	+
TH-Nov	0.121	-	323.7	7.77	+
	0.479	-	332.5	18.4	+
	0.536	-	353.1	43.3	++
	0.683	-	332.5	18.4	+
	0.758	-	332.3	18.2	+

**Table 5:** Honey samples.

Bee Species	Sample	Harvest Date
*Tetragonula carbonaria*	C1	May 2022
	C2	
	C3	
	C4	
	C5	
	C6	
	C7	
	C8	
	C9	
	C10	
*Tetragonula hockingsi*	H1	May 2022
	H2	
*Tetragonula carbonaria*	C13	September 2022
	C14	
	C15	
	C16	
	C17	
	C18	
	C19	
	C20	
*Tetragonula hockingsi*	H5	September 2022
	H6	
*Tetragonula carbonaria*	C21	November 2022
	C22	
	C23	
	C24	
	C25	
	C26	
	C27	
	C28	
*Tetragonula hockingsi*	H7	November 2022
	H8	

**Table 6:** Honey blends.

Honey Blend	Bee Species	Harvest Date
TC-May (*n* = 10)	*Tetragonula carbonaria*	May 2022
TH-May (*n* = 2)	*Tetragonula hockingsi*	May 2022
TC-Sep (*n* = 8)	*Tetragonula carbonaria*	September 2022
TH-Sep (*n* = 2)	*Tetragonula hockingsi*	September 2022
TC-Nov (*n* = 8)	*Tetragonula carbonaria*	November 2022
TH-Nov (*n* = 2)	*Tetragonula hockingsi*	November 2022

**Table 7:** Categories of antioxidant activity for individual bands based on DPPH % RSA.

% DPPH RSA	Category	Inference
0.0%	0	No activity
1.0–33.3%	+	Low activity
33.4–66.6%	++	Medium activity
66.7–100.0%	+++	High activity

## Data Availability

The original contributions presented in this study are included in the article; further inquiries can be directed to the corresponding author.

## Supplementary Materials

The following supporting information can be downloaded at https://www.mdpi.com/article/10.3390/molecules30061223/s1: Figure S1: HPTLC chromatograms of honey samples TC-May (a), TH-May (b), TC-Sep (c), TH-Sep (d), TC-Nov (e), and TH-Nov (f), using the mobile phase toluene/ethyl acetate/formic acid (6:5:1). Chromatograms obtained prior to derivatisation at 254 and 366 nm (A), after derivatisation with VSA at 366 nm and white light (B), and after derivatisation with NP-PEG at 366 nm (C).
