# Supplementary material for: Physicochemical Characteristics, Antioxidant Properties, and Identification of Bioactive Compounds in Australian Stingless Bee Honey Using High-Performance Thin-Layer Chromatography
Source: Molecules. 2025 Mar 9;30(6):1223. doi: 10.3390/molecules30061223 (PMC11944790; doi:10.3390/molecules30061223)
Supplement: Supplementary file 1 [file molecules-30-01223-s001.zip › molecules-3487351-supplementary.pdf]

Supplementary Material:

Physicochemical Characteristics, Antioxidant Properties and Bioactive compounds in Australian Stingless Bee Honey

(a)

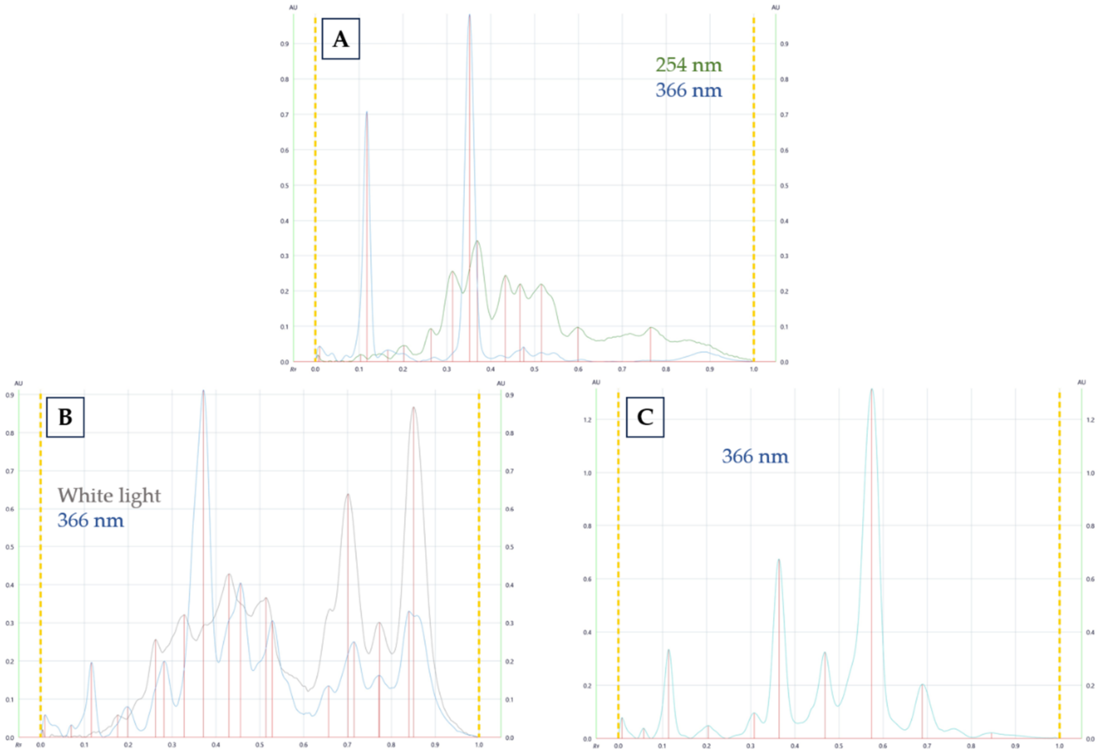

(b)

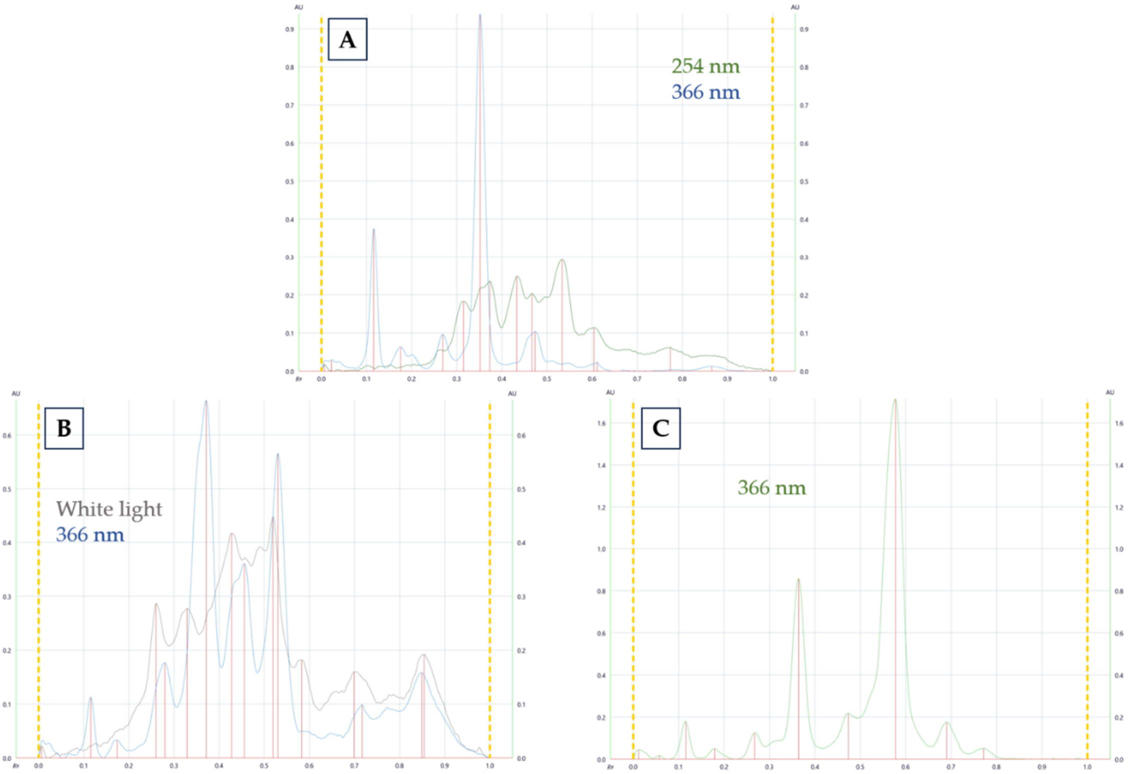

(c)

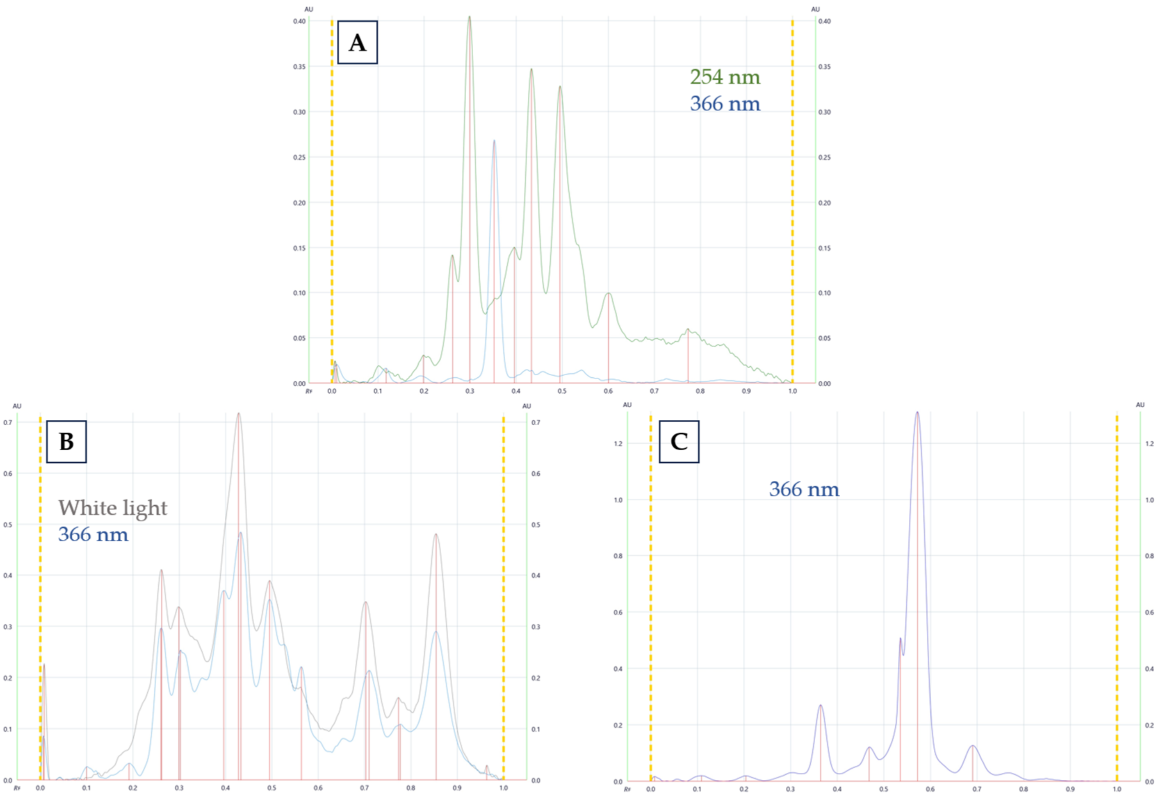
A
254 nm
366 nm
B
White light
366 nm
C
366 nm

(d)

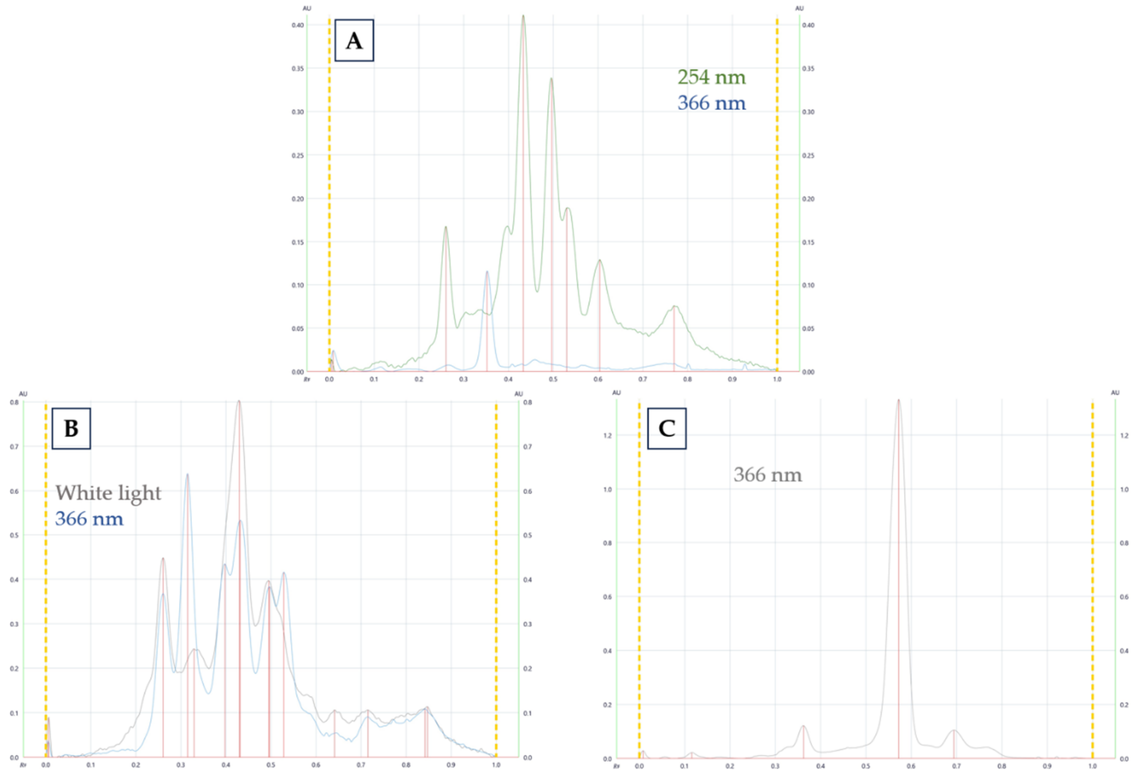
A
254 nm
366 nm
B
White light
366 nm
C
366 nm

(e)

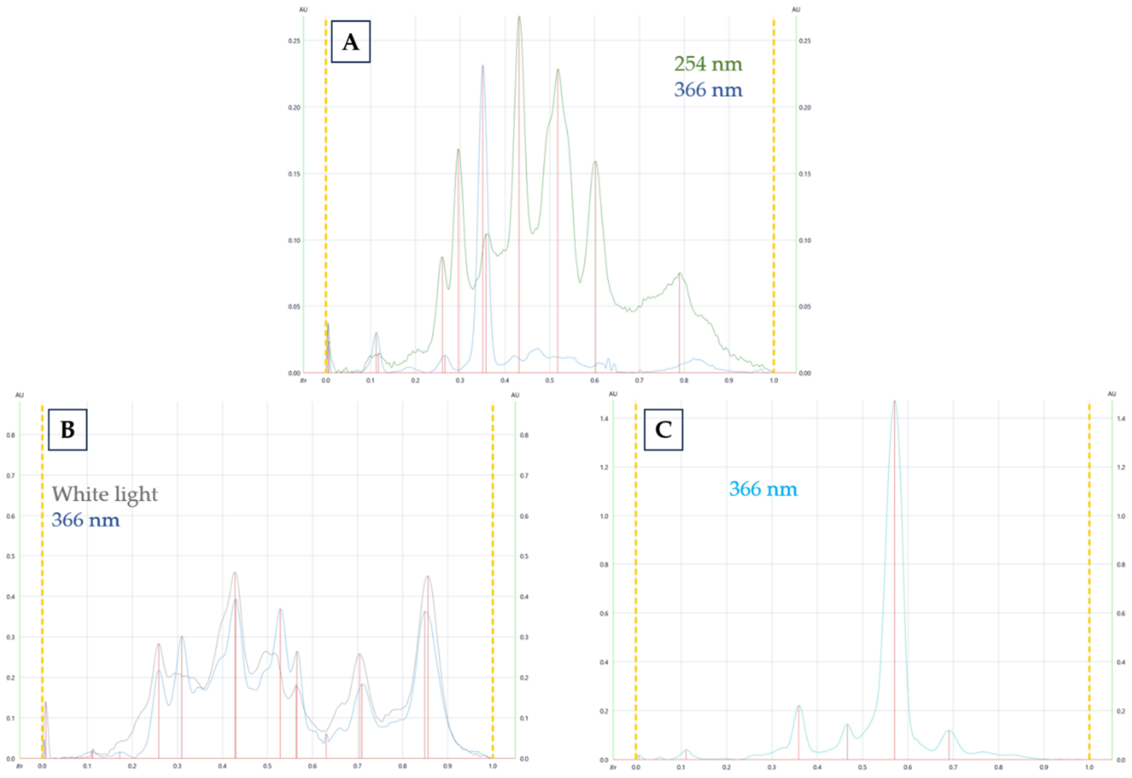

(f)

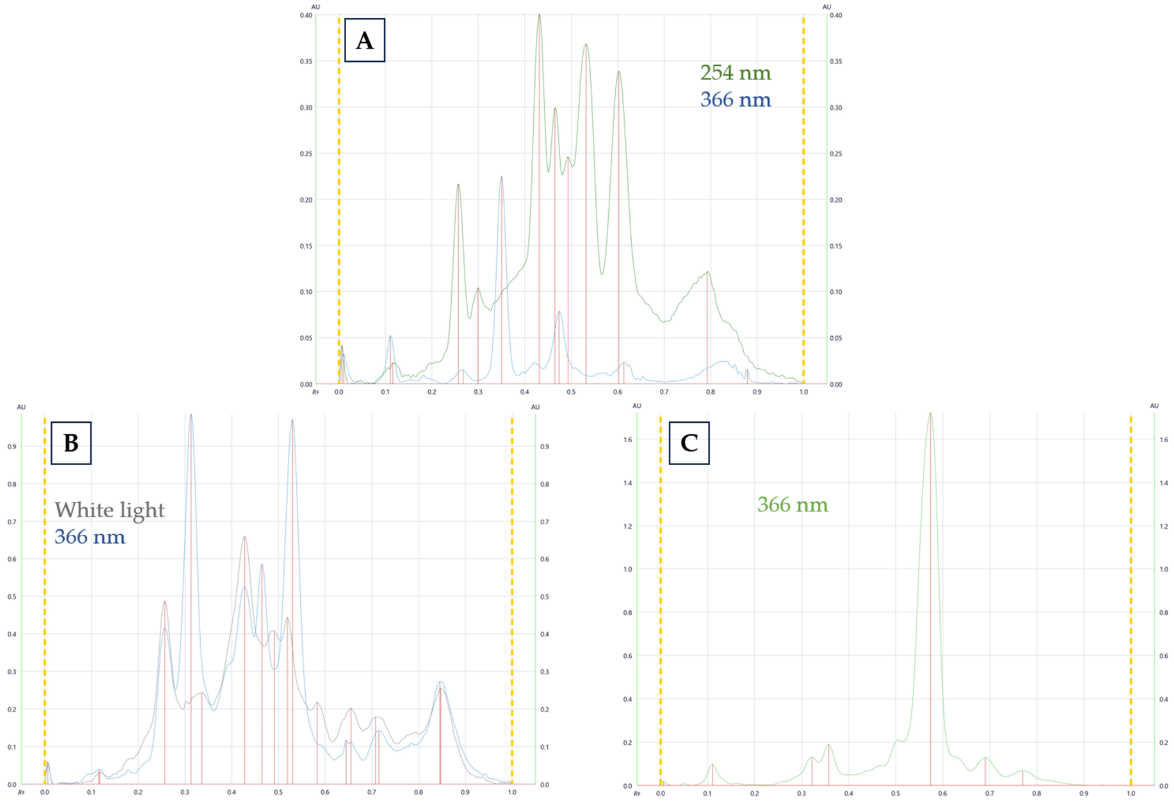

Figure S1. HPTLC chromatograms of honey samples TC-May (a), TH-May (b), TC-Sep (c), TH-Sep (d), TC-Nov (e), and TH-Nov (f) using mobile phase toluene:ethyl acetate:formic acid (6:5:1). Chromatograms obtained prior to derivatisation at 254 and 366 nm (A), after derivatisation with VSA at 366 nm and white light (B) and after derivatisation with NP-PEG at 366 nm (C).
